# Executive and motivational processes in adolescents with Attention-Deficit-Hyperactivity Disorder (ADHD)

**DOI:** 10.1186/1744-9081-1-8

**Published:** 2005-06-27

**Authors:** Maggie E Toplak, Umesh Jain, Rosemary Tannock

**Affiliations:** 1 Brain and Behaviour Research Program, Research Institute, The Hospital for Sick Children, Toronto, Canada; 2 York University, Toronto, Canada; 3 Centre for Addiction and Mental Health, Toronto, Canada; 4 Centre for Advanced Study at the Norwegian Academy of Science and Letters 2004–2005

## Abstract

**Background:**

The objective of the current study was to examine performance and correlates of performance on a decision-making card task involving risky choices (Iowa Gambling Task) in adolescents with ADHD and comparison controls. Forty-four participants with ADHD and 34 controls were administered measures of estimated intellectual ability, working memory, and the card task. Also, behavioural ratings were obtained from parents and teachers.

**Results:**

Adolescents with ADHD scored lower on the measures of intellectual ability, working memory, and made less advantageous selections on the card task compared to controls. Performance on measures of intellectual ability and working memory were unrelated to card task performance in both the ADHD and control samples. Parent ratings of hyperactivity/impulsivity were significantly associated with card task performance in the adolescents with ADHD, but not in controls.

**Conclusion:**

These findings demonstrate impaired decision-making in adolescents with ADHD, and the separability of motivational and executive function processes, supporting current dual pathway models of ADHD.

## Background

Individuals with Attention-Deficit-Hyperactivity Disorder (ADHD) are more likely than their peers to make poor real-life decisions, as these individuals are described as impulsive [1], engage in more risky activities than controls [2,3], and tend to exhibit a preference for immediate rather than delayed rewards [4-7]. Much of the recent work over the last 20 years in ADHD has focused on the cognitive features of ADHD, defined as executive functions [8-10]. However, recent theories of ADHD have included both executive processes and motivational style characterized by delay aversion as two important pathways in ADHD [11,12]. The purpose of this study was to elaborate and extend this conceptualization by examining performance on a risky-choice decision-making card task, also known as the Iowa Gambling Task, in a sample of adolescents with ADHD and comparison controls. We refer to this task as the card task for simplicity. In addition, we were also interested in examining associations between performance on the card task and measures of intellectual ability and working memory.

The most recent development in the field has been an emphasis on multiple pathways for explaining impairment in ADHD [13-16,12]. Sonuga-Barke's [12] most recent model of ADHD argues for a dual pathway model of ADHD, highlighting both the executive and motivational, delay aversion aspects of ADHD. Specifically, the executive pathway involves a dysregulation of thought and action that is primarily characterized by a core deficit in inhibitory control [11]. Alternatively, the motivational pathway is hypothesized to mediate a link between behavioural symptoms, task engagement, and a biologically embedded alteration in reward mechanisms [11]. One important change reflected in this dual-pathway conceptualization is that the executive and motivational pathways are not regarded as competing theories, rather that deficits in both processes are thought to give rise to the manifestation of ADHD [12]. Sonuga-Barke [11] argues that these two pathways likely give rise to an ADHD diagnosis of the Combined subtype, and that the executive pathway is more likely associated with more severe and generalized cognitive impairment. This idea of dual pathways maps well onto clinical and research characterizations of ADHD, as the Inattentive subtype has been associated with executive dysfunction [17] and the Hyperactive/Impulsive subtype has been associated with the impulsiveness feature of ADHD [18]. Sonuga-Barke, Dalen, & Remington [19] reported that executive function and delay aversion made significant, independent contributions to ADHD symptoms in a sample of children. Similar findings have also been reported by Crone, Jennings, and van der Molen [20].

In addition to separability at a neuropsychological level, it has been hypothesized that the executive and motivational pathways are rooted in conceptually similar, but functionally segregated brain circuits [12]. It has been hypothesized that the executive pathway receives inputs from the dorsolateral prefrontal cortex to the dorsal portion of the neostriatum, as well as reciprocal connections from subcortical regions, including the dorso-medial sections of the thalamus. Alternatively, the motivational pathway centers on the reward circuits of the ventral striatum, specifically the nucleus accumbens, with connections from frontal regions, including the anterior cingulate and orbitofrontal cortex, and the amygdala. Importantly, this dual pathway model offers a theoretical account of interactions between cortical and subcortical pathways in the regulation of executive processes and motivation. The notion of separable pathways has also been theorized at a neurobiological level as well. Sagvolden et al.'s [21] dynamic developmental model of ADHD suggests that altered dopamine branches give rise to the different ADHD symptomotology, specifically, a hypofunctioning mesolimbic dopamine branch gives rise to delay aversion, a hypofunctioning mesocortical dopamine branch gives risk to poor executive functions, and a hypofunctioning nigrostriatal dopamine branch gives rise to other motor symptoms. This neurobiological account is consistent with the idea of separable pathways in ADHD, specifically the executive and delay aversion pathways. It is important to understand and test the relative contribution of these different pathways to the clinical manifestation of ADHD at both behavioural and neurobiological levels of analysis.

An important behavioural measure, called the Iowa Gambling Task (or card task), was designed to simulate the uncertainties of real-life decision-making, necessitating the weighting of rewards and penalties. In this task, participants are asked to select cards from four decks, which unbeknownst to these participants vary on expected outcome [two decks are composed of quick high gains and high losses (disadvantageous decks) or low gains and low losses (advantageous decks)] and in frequency of penalties (two decks have frequent, smaller penalties, while the other two decks have infrequent, large penalties). Expected outcome and frequency of penalties are crossed, creating four different conditions with these four decks. What has been particularly striking and important about these studies on Iowa Gambling Task performance with patients who have ventromedial cortex lesions is not simply the link between brain function and higher cognitive processes, but also the possibility that this form of reasoning may be fairly modular and localized, and separable from other cognitive abilities, such as intelligence [22].

The card task was originally used to study patients with ventromedial cortex lesions [22]. The ventromedial cortex, which may include the orbitofrontal cortex, but some have argued for subtle distinctions between these areas [23,24]. For present purposes, the orbitofrontal cortex and ventromedial areas have generally been implicated in the emotional experience associated with gains and losses in decision processes, which is to be differentiated from other frontal processes and regions, including the dorsolateral prefrontal cortex and anterior cingulate [25].

Patients with ventromedial prefrontal cortex lesions displayed less optimal performance than normal controls, making considerably more selections from the disadvantageous decks than from the advantageous decks [26]. This link between the orbitofrontal cortex and impulsivity has been described previously by others, such as, Newman et al. [18], who reported on brain lesion studies with rats which suggested an association between disinhibitory syndromes in humans (including, psychopathy, addictions, and ADHD) and lesions in the orbitofrontal cortex. Other clinical samples have reportedly displayed less optimal performance on the card task, including high school students with multiple suspensions [27], heroin addicts [28], and individuals with antisocial behaviour and psychopathic tendencies [29]. Sex differences have also been reported on card task performance, with females tending to make more selections from disadvantageous decks than males [30].

Performance on the card task has also been examined in a small group of adults with and without ADHD [31] and in a sample of adolescents with disruptive behaviour disorders, including ADHD and conduct problems [32]. In the study which included adolescents with disruptive behaviour disorders [32], adolescents with disruptive behaviour disorders displayed less optimal performance on this task than controls. In the study using adults with ADHD [31], no behavioural differences were observed on decision-making performance. However, PET scans revealed that brain activation in the ADHD group was less extended than in the control group. Specifically, control participants recruited hippocampal and insular regions more than adults with ADHD, and the adults with ADHD engaged the caudal part of the right anterior cingulated more than the controls. These results, therefore, suggest that this task activates different processes and brain regions. The behavioural significance of these differential activations is unclear at this time, and suggests that more work needs to be done on elaborating our understanding of the motivational pathway [14]. The card task and its links with patients with ventromedial lesions makes it an important task to further study and understand the motivational pathway in ADHD.

As recent models of ADHD suggest two potential pathways supporting the motivational and executive processes in ADHD [12], we must also investigate the possible associations and/or dissociations between these processes. One study investigated card task performance and working memory in normal controls, patients with lesions in the ventromedial prefrontal cortex, and patients with lesions of the dorsolatoral/mesial region of the prefrontal cortex [33]. Bechara et al. [33] reported a cognitive and anatomical double dissociation between card task performance and working memory. Others have also reported a dissociation between impulsivity and working memory in the orbitofrontal and dorsolateral regions of the prefrontal cortex [34]. However, Ernst et al. [32] found that IQ was a significant predictor of card task performance in their sample of adults with substance abuse disorders and adolescents with behaviour disorders. Hinson, Jameson, & Whitney [35] reported that increasing working memory load resulted in poorer performance on the card task. Therefore, mixed results have been reported on the behavioural associations between card task performance and working memory. These findings suggest that lesions in the ventromedial cortex may not uniquely or independently explain risky decision-making, and that contributions from other regions, such as the frontostriatal dopamine system [25], and other processes, such as working memory [35,36], may also play a role in decision-making involving gains and losses. Therefore, we also examined the relationship between card task performance and intellectual ability, and working memory in our sample of adolescents with ADHD.

The purpose of the present study was to examine how adolescents with ADHD weight risks and benefits in the card task compared to comparison controls. Impaired performance on the card task would importantly implicate ventromedial prefrontal regions for study in ADHD, which would importantly extend the motivational pathway of the dual pathway model of ADHD [11,12] by suggesting a link between subcortical structures, like the nucleus accumbens, and cortical structures, like the ventromedial cortex. In addition, associations between card task performance, intellectual ability, and working memory were also examined. A dissociation between performance on the card task and intellectual ability and working memory (our executive tasks) was predicted, which would be consistent with dual pathway models of ADHD.

## Results

### Data Analysis and Statistical Methods

Card Task Analysis. We conducted an analysis of covariance (ANCOVA), with outcome (advantageous vs. disadvantageous) and frequency (frequent vs. infrequent penalties) as within-subject factors and group (ADHD vs. control) as a between subject factor. Gender, FSIQ, auditory working memory, and visual-spatial working memory were examined as covariates. All posthoc analyses were a priori planned comparisons, and were conducted using the Bonferroni correction [37]. Then, correlational analyses were conducted separately and together in the ADHD and control samples to examine whether estimated FSIQ, or working memory were significantly correlated with card task performance; this provided a further, converging examination of the relationship between card task performance, FSIQ, and working memory. A repeated measures analysis was conducted to examine learning across trials on the card task. An ANCOVA analysis was conducted to examine the impact of subtype in the ADHD sample, covarying for gender. We also examined correlations between card task performance and behavioural ratings by parents and teachers.

### Group Differences on Standardized Measures and Behaviour Ratings

Table 1 displays the standardized measures and clinical characteristics of the adolescents with ADHD and comparison controls. Overall, adolescents with ADHD displayed significantly lower scores, albeit in the normal range, on the measures of FSIQ, and auditory and visual-spatial working memory than comparison controls.

**Table 1:** Diagnostic Characteristics of the ADHD and Comparison Control Groups

	ADHD	n	Controls	n	F (1, 77)
Age	15.6 (1.4)	44	15.4 (1.5)	34	0.24
**Standardized Measures**					
Estimated VIQ	105.5 (10.6)	44	109.9 (10.0)	34	3.48
Estimated PIQ	101.6 (11.2)	44	106.4 (11.3)	34	3.45
Estimated FSIQ Score	104.1 (10.1)	44	109.4 (10.2)	34	5.20*
Auditory Working Memory Scaled Score	9.6 (3.5)	44	11.9 (2.5)	34	10.35**
Visual-Spatial Working Memory Scaled Score	9.2 (3.4)	44	11.3 (2.7)	34	8.41**
**Diagnostic Characteristics from Conners Ratings**					
*Parent*					
Inattention T-score	73.2 (10.1)	43	49.3 (6.8)	32	134.25***
Hyperactivity/Impulsivity T-score	72.7 (12.0)	43	51.6 (7.8)	32	74.85***
*Teacher*					
Inattention T-score	70.9 (14.8)	37	49.7 (9.9)	28	42.97***
Hyperactivity/Impulsivity T-score	65.4 (16.3)	37	51.1 (9.2)	28	17.29***

*** p < .001, **p < .01, *p < .05

### Group Differences on Card Task

The means and standard deviations for all 100 trials of the card task for each deck are presented in Table 3. Using an analysis of covariance (ANCOVA), we found that outcome was significant, *F*(1, 76) = 12.45, *p *= .001, indicating that more cards were selected overall from the disadvantageous than the advantageous decks by all participants. Frequency was also significant, *F*(1, 76) = 33.94, *p *= .0001, indicating that all participants selected more cards from the infrequent penalty decks than the frequent penalty decks. The outcome by group and frequency by group interactions were non-significant. The outcome by frequency interaction was significant, *F*(1, 76) = 4.47, *p *= .038, indicating that most cards were selected from deck B, *p = .0001*. The outcome by frequency by group interaction was significant, *F*(1, 76) = 5.76, *p *= .021. Posthoc analyses indicated that participants with ADHD selected more cards from deck B, *p = .045*, and significantly fewer cards from deck D, *p = .018*, than comparison controls. The group main effect was not significant. Sex, FSIQ, auditory working memory, and visual-spatial working memory did not enter as significant covariates.

**Table 3:** Mean scores of the adolescents with ADHD and comparison controls for the last 100 selections on the card task (standard deviations in parentheses)

Deck	ADHD	n	Control	n	F(1,77)	Cohen's d
Deck A – Disadvantageous	23.3 (9.7)	44	24.4 (7.3)	34	0.34	-0.13
Deck B – Disadvantageous	34.1 (10.0)	44	29.4 (10.4)	34	4.17*	0.46
Deck C – Advantageous	19.9 (8.3)	44	20.3 (8.8)	34	0.06	-0.05
Deck D – Advantageous	21.6 (7.0)	44	26.9 (12.2)	34	5.81*	-0.53
Monetary Outcome	-10.0 (7.0)	44	-6.4 (11.4)	34	2.99†	-0.38

* p < .05, † p < .10

Correlational analyses with decks B and D were also conducted for a convergent analysis. Correlational analyses were conducted separately within the ADHD and control groups, as it is important to determine whether these variables co-vary differently in the ADHD and control groups. There were no significant associations between deck selections on the card task and intellectual ability or working memory in the ADHD or control samples. When this same analysis was conducted within the entire sample, the same results were obtained. Effect sizes in Table 3 indicate moderate effects for selections from decks B and D. Participants with ADHD also tended to lose more money than controls, an effect which was marginally significant between groups.

**Figure 1:**
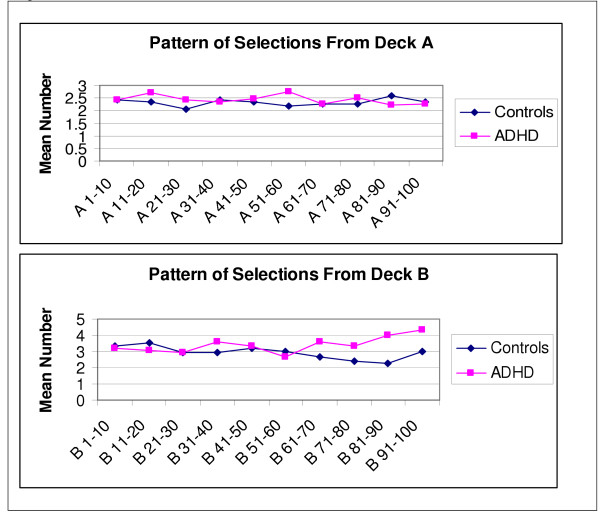
Pattern of Selections on the Card Task from Decks A and B.

**Figure 2:**
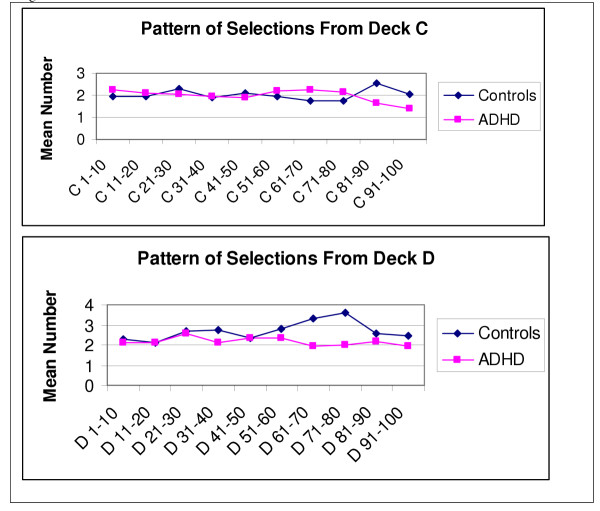
Pattern of Selections on the Card Task from Decks C and D.

Figures 1 and 2 display a visual breakdown of the mean number of cards that were selected from each deck at every ten card interval. The first 50 trials for both ADHD and control groups do not suggest any pattern in card selections, suggesting a sampling and learning phase in both groups. As evidenced in the group differences, the trends differ for decks B and D in the ADHD and control groups for the last 50 card selections. Clearly, the ADHD group continues to select more cards from deck B right until the end of the game, whereas controls display a clear preference for selecting cards from deck D. Selections from deck C slightly mirror those of deck D, but there were obviously no differences evident in the selections from deck A. We conducted analyses of the last 50 card selections for decks B and D. Using a 2 (group: ADHD vs. controls) X 5 (Block: B51-60, B61-70, B71-80, B81-90, B91-100) repeated measures MANOVA to examine the last 50 card selections from deck B, we obtained a significant group by block interaction, *F*(1, 76), 4.33, *p *= .041, indicating that adolescents with ADHD picked progressively more cards from deck B than controls. When this same analysis was conducted with deck D, the interaction did not reach significance.

The open-ended question about the content of the decks was coded for whether participants recognized that some decks were more advantageous than others. It was found that 58% (n = 23) and 86% (n = 36) of the adolescents with ADHD recognized that decks A and B were disadvantageous, and 89% (n = 34) and 81% (n = 34) recognized that decks C and D were advantageous. Of the controls, 79% (n = 23) and 91% (n = 30) recognized that decks A and B were disadvantageous, and 83% (n = 20) and 87% (n = 27) recognized that decks C and D were advantageous. One participant with ADHD (2 %) ended the game with a net gain, and 10 control participants (29 %) ended the game with a net gain. Overall, these data indicate that participants in both groups generally understood the task and recognized the advantageous and disadvantageous decks. One exception to this is that only 58% of the adolescents with ADHD recognized that A was a disadvantageous deck, supporting the idea that the content of this deck was more difficult to track.

### Subtype Effects and Correlational Analyses Between The Card Task and Behavioural Measures

Twenty-seven of our adolescents with ADHD met criteria for the Combined subtype, and 17 met criteria for the Inattentive subtype. We examined the effect of subtype on card task performance within the ADHD group using a multivariate ANOVA, with subtype (Inattentive vs. Combined) as a between-subject factor and outcome (advantageous vs. disadvantageous decks) and frequency (frequent vs. infrequent penalties) as within-subject factors. The subtype by frequency interaction was significant, *F*(1, 42) = 4.41, *p *= .042. Posthoc analyses indicated that participants with ADHD of the Combined subtype, *M *= 57.9, *SD *= 9.1, tended to select more cards from decks B and D than participants with ADHD of the Inattentive subtype, *M *= 52.2, *SD *= 8.3, *p *= .043. Then, participants with ADHD of the Inattentive subtype, *M *= 47.8, *SD *= 8.3, tended to select more cards from decks A and C than participants with ADHD of the Combined subtype, *M *= 42.0, *SD *= 9.1, *p *= .041. We examined sex as a covariate of subtype, but no significant associations were obtained. Five of the females with ADHD met criteria for the Combined subtype, whereas only one met criteria for the Inattentive subtype.

With the behavioural measures, two associations between hyperactive/impulsive symptoms on the K-SADS-PL (that is, DSM-IV-TR symptoms), *r *= .31, *p *= .05, and Conners' parent rating of hyperactivity/impulsivity, *r *= .31, *p *= .04, correlated significantly with the total number of card selections from disadvantageous deck B in the ADHD sample, however, there were no such associations within the control sample. The associations between inattentive symptoms and deck selections did not reach significance in the ADHD or control samples. These correlations are displayed in Table 4. When the same correlations were conducted with the entire sample, the significant association between deck B selections and parent report of hyperactivity/impulsivity on the Conners'scales remained, *r *= .25, *p *= .031.

**Table 4:** Correlations between Card Task Selections and Behaviour Rating Measures in Adolescents with ADHD and Controls

		Deck B (100 Trials)	Deck D (100 Trials)
**Behaviour Rating Measures**			
K-SADS – Number of Inattentive Symptoms	Control (n = 0)	--	--
	ADHD (n = 42)	-.22	.23
K-SADS – Number of Hyperactive/Impulsive Symptoms	Control (n = 0)	--	--
	ADHD (n = 44)	.31*	.02
Conners Parent Inattention T-Score	Control (n = 34)	.26	.00
	ADHD (n = 43)	-.10	.29
Conners Parent Hyperactivity/Impulsivity T-Score	Control (n = 34)	-.22	.12
	ADHD (n = 43)	.31*	.26
Conners Teacher Inattention T-Score	Control (n = 28)	-.07	.28
	ADHD (n = 37)	-.28	-.06
Conners Teacher Hyperactivity/Impulsivity T-Score	Control (n = 28)	-.13	.09
	ADHD (n = 37)	-.11	.14

*p < .05

## Discussion

The results of the present study indicated that the adolescents with ADHD made less optimal selections on the card task than controls by selecting more cards from the disadvantageous deck B and fewer cards from the advantageous deck D than controls. Adolescents with ADHD also scored lower on estimated FSIQ, and auditory and visual-spatial working memory than controls, but these variables were not significant covariates of card task performance. In the correlational analyses with the behavioural ratings, it was parent report of hyperactivity and impulsivity, not estimated FSIQ or working memory, which was associated with performance on the card task in the adolescents with ADHD. Sex did not enter as a significant covariate of card task performance.

Consistent with previous studies, our participants with ADHD displayed a pattern of performance similar to adolescents with behaviour disorders [32]. In particular, the group differences in the current study emerged on only two of the decks; importantly, these two particular decks (decks B and D) necessitated less tracking of expected value as penalties were less frequent. This finding suggests that the frequency of dispensing rewards and penalties should be considered as a potential variable in this task, and must be examined systematically. It may be the case that requiring participants to track more carefully may result in the recruitment of executive processes, such as working memory [35]. Overall, the group differences on the card task highlight poor behaviour regulation and impulsivity as critical features in the profile of ADHD, which have both been described as pervasive features of ADHD [1].

In addition, we did not find any association between selections on the card task and on our measures of estimated FSIQ or working memory, suggesting that these processes are behaviourally separable. While our adolescents with ADHD displayed lower estimated FSIQ and working memory scores than controls, our ANCOVA and correlational analyses suggest that their less optimal performance on the card task is not attributable to limitations in FSIQ or working memory, which is consistent with the findings of Bechara et al. [33] and Berlin et al. [34], but not with Ernst et al. [32] or Hinson et al. [35]. Importantly, Ernst et al. [32] used a more heterogeneous sample than we used, including adolescents with conduct problems and adults with substance abuse problems. Then, as Hinson et al. [35] increased memory load on the card task, it would be expected that further taxing of working memory during administration of the card task would negatively affect performance. Notably performance on both estimated FSIQ and working memory in our study were not associated or correlated with card task performance. Cognitive scientists understand tests of cognitive and intellectual ability as general indicators of cognitive efficiency [44,47-49], which is consistent with the general concept of executive functions [8]. Indeed, correlations between estimated FSIQ and the working memory measures within the ADHD and control samples ranged from *r *= .35, *p *= .045 to *r *= .57, *p *= .0001. Therefore, estimated FSIQ and our working memory measures provide a reasonable index of executive, dorsolateral types of processes.

These findings are consistent with separable pathway models of ADHD [15,11,19,21], which includes the separable contributions of both executive processes and behavioural regulation variables. The motivational pathway highlights the impulsive tendencies [1], delay aversion [5-7], and sensitivity to rewards and response costs [50-52] in ADHD. In addition, the current study also extends the conceptualization of the motivational pathway to include cortically-based processes in ADHD, particularly the ventromedial prefrontal cortex [19], suggesting that more research is needed to better parse how frontal and subcortical mechanisms function and interact in the clinical manifestation of ADHD. These findings are also consistent with cognitive developmental models of normative development which include the separable contributions of "hot" affective decision-making versus "cool" executive function processes [53].

Studies have consistently replicated the role of executive function deficits in ADHD [8-10], however, further elaboration and understanding are needed at a behavioural level of analysis for what has been termed as the motivational pathway in ADHD. Drawing from the work by Damasio's interpretation of card task performance [54-56], one possibility worthy of further investigation for less optimal card selections in the ADHD group is dysregulation of somatic markers. It may be the case that individuals with ADHD have weaker somatic or physiological cues to guide risky choices, which would be consistent with Damasio's [54-56] somatic marker hypothesis. Somatic markers, or emotions, assist by constraining the decision-making space, giving various alternatives preferential availability over other alternatives [57], and serve an adaptive evolutionary human function, consistent with cognitive science perspectives on the role of emotion [58]. Damasio [54] argues that his patients with ventromedial lesions lack the physiological cues needed to signal risky choices, as evidenced by skin conductance studies performed in his lab [26,59]. At least two studies have been conducted investigating the physiological reactions of children with ADHD and controls in the presence of reward and extinction conditions [60]. It was reported that children with ADHD displayed a faster heart rate habituation to reward and less of a galvanic skin response during habituation than controls. A study by Crone, Jennings, and van der Molen [20] found that children with ADHD had lower heart rate responses to immediate reward feedback than comparison controls, but no group differences were observed in skin conductance responses. These studies suggest that individuals with ADHD may experience a different physiological reaction than controls in the presence of rewards, suggesting that these cues may give way to different somatic markers, affecting decision-making in these individuals. This is a viable hypothesis in individuals with ADHD that deserves further study and consideration for understanding the motivational pathway. Notably, any difficulties in the physiological and/or affective regulation are likely to be more subtle in individuals with ADHD than in patients with ventromedial lesions, which is one reason why the statistical analyses may not have yielded such strong findings. Other limitations of the present study include sample heterogeneity, the gender imbalance between groups, and a lack of a full psychiatric diagnostic assessment in the control participants. Therefore, future research and methodologies should take these variables into account.

Another critical finding in this study was that card task performance was correlated with the behavioural and diagnostic symptoms of hyperactivity and impulsivity, but not with inattention, estimated FSIQ, or working memory. This correlation is consistent with Dinn, Robbins, and Harris [61], who found that adults with ADHD – Combined Subtype performed worse on orbitofrontal tasks, whereas adults with ADHD-Inattentive subtype performed worse on dorsolateral tasks. Motivational regulation difficulties seem to be associated with the hyperactive and impulsive features of ADHD [18], and the executive function deficits are associated with the inattentive features of ADHD [17], which is consistent with the current conceptualization. While subtype did not differentiate performance on advantageous or disadvantageous card selections, this may have been due to the fact that we did not have any participants with only the Hyperactive/Impulsive subtype. The next step will be to understand the relationships between other cognitive processes thought to be deficient in ADHD. For example, the relationship between inhibitory control [9], other components of inhibition [62], and time perception [63], with other executive and motivational processes as defined in the dual pathway model [12], and to examine how they interact and give rise to the clinical presentation of ADHD.

## Conclusion

The results of the current study demonstrated that adolescents with ADHD displayed impaired performance on the Iowa Gambling card task as well as on measures of intellectual ability and working memory in comparison to adolescent controls. Notably, performance on the card task and the measures of intellectual ability and working memory were not associated in the ADHD and control samples. Non-optimal card selections were associated with hyperactive and impulsive symptoms in the adolescents with ADHD. These findings provide support for the separable motivational and executive pathways in current models of ADHD [11,12]. Further research must elaborate what processes constitute the motivational pathway in ADHD, and how these processes may interact with executive processes and give rise to the clinical presentation of ADHD.

## Method

### Participants

Two groups of adolescents participated: 44 adolescents (86% male) with a confirmed clinical diagnosis of ADHD based on DSM-IV criteria and 34 comparison adolescents (41% male). Of our ADHD sample, 45% (n = 20) met criteria for Predominantly Inattentive subtype, and 55% (n = 24) met criteria for Predominantly Combined subtype. All adolescents were between the ages of 13 and 18 years of age (*M *= 15.5; *SD *= 1.5). Adolescents with ADHD were recruited from the YEARS (Youth, Education, and Assessment Research Service) Program. Adolescents in the control comparison group were recruited through hospital staff and community resources. All adolescents participating in the study were native English speakers. Adolescents were excluded if they had evidence of psychosis, pervasive developmental disorder, a serious medical condition, or an estimated Full-Scale IQ (FSIQ) below 80.

ADHD Sample. All adolescents had a DSM-IV diagnosis of ADHD confirmed by a systematic and comprehensive clinical diagnostic assessment conducted at the time of the study. The assessment comprised a semi-structured clinical diagnostic interview [Schedule for Affective Disorders and Schizophrenia for School-Age Children-Present and Lifetime Version; K-SADS-PL; [38]]. Also, parents and teachers completed the Conners' Rating Scales-Revised [39] to obtain standardized ratings of behaviour. Diagnosis of ADHD in adolescents was based on the following algorithm: 1) met DSM-IV criteria according to the clinician summary based on the K-SADS-PL interviews; and 2) met the clinical cut-offs for inattentive or hyperactive/impulsive symptoms on the Conners teacher questionnaires (t-score > 70) in order to ensure pervasiveness of symptoms across settings. The K-SADS was conducted separately with the adolescent and parent, and the clinician summarized the information from both informants. The K-SADS interview was the primary source for diagnosis, and the Conners' scales did not always reach the threshold t-score, particularly on the teacher reports. If parents reported a history of ADHD symptoms (both at home and from school reports) and evidence of pervasiveness across settings during the K-SADS interview, this information was adequate for a diagnosis of ADHD.

Subtypes were determined by counting the number of inattentive and/or hyperactive/impulsive symptoms on the K-SADS, and comparing with the Conners scales for convergence. Participants needed a total of six symptoms of inattention endorsed to be identified as the Inattentive subtype, six symptoms of hyperactivity or impulsivity to be identified as the Hyperactive/Impulsive subtype, or six of each to be identified as the Combined subtype. A reading or math learning disability was defined as a score below the 25^th ^percentile on a measure of reading or math achievement. Many of the adolescents with ADHD had comorbid disorders: 16 (36%) had a Learning Disability, 12 (27%) had Oppositional Defiant Disorder, four had an Anxiety Disorder, three had Depression, and one had Conduct Disorder. Diagnostic characteristics of the sample are presented in Table 1.

Fourteen of our participants used stimulant medication (30%), eight had previously used stimulant medication (17%), five used a non-stimulant medication, and 18 had never used psychoactive medication (43%). All adolescents were asked to stop taking any medication for six half-lives prior to assessment, except for the two participants who used antidepressants.

Comparison control sample. The Conners' questionnaires were given to parents and adolescents to screen for any mental health concerns. Any participants who obtained scores above a t-score of 60 were interviewed further with a complete K-SADS-PL interview to rule out any diagnosis of ADHD. A total of four K-SADS-PL were conducted to follow up on issues raised on the Conners questionnaires in the comparison control group. Otherwise, a psychiatric assessment with control participants was not completed.

### Standardized Tasks

Intellectual Ability. The Wechsler Abbreviated Scale of Intelligence [WASI; [40]] comprised four subtests (Vocabulary, Block Design, Similarities, and Matrices), and was used to provide an estimate of verbal (VIQ), nonverbal (PIQ), and Full-Scale (FSIQ) intellectual ability.

Auditory and Visual-Spatial Working Memory. We used two different tasks to measure memory performance, one auditory-verbal and one visual-spatial. Our measure of auditory memory was the Digit Span subtest (Forwards and Backwards components) from the Wechsler Intelligence Scale for Children – Third Edition [WISC-III; [40]] and the Wechsler Adult Intelligence Scale – Third Edition [WAIS-III; [42]]. Our measure of visual-spatial memory was the Spatial Span subtest (Forwards and Backwards components) from the WISC-III Processing Instrument [WISC-III-PI, [43]]. The combination of the forwards and backwards components of each memory measure provide a composite of maintenance and working memory [44]; for simplicity, we have termed these as our working memory measures.

### Experimental Task

Card Task. This task was designed after the Iowa gambling task by Bechara et al. [26]. The materials required for this task included: four decks of cards designed by the researcher, monopoly play money, and a hand counter to track the number of trials during the task. Four decks of cards were placed in front of the participant, labeled as deck A, B, C, or D. On the back of each card, there was either a reward or a reward and penalty indicated. Each deck varied on expected outcome [two decks are composed of quick high gains and high losses (disadvantageous decks) or low gains and low losses (advantageous decks)] and in frequency of penalties (two decks have frequent, smaller penalties, while the other two decks have infrequent, large penalties). Expected outcome and frequency of penalties are crossed, creating four different conditions with these four decks. Therefore, deck A was a disadvantageous deck with frequent, lower penalties, deck B was a disadvantageous deck with infrequent, high penalties, deck C was an advantageous deck with frequent, lower penalties, and deck D was an advantageous deck with infrequent, high penalties. The cards were organized in the exact same order for each and every participant. There were 50 cards in each deck. The reward-penalty schedules that were used for this task are displayed in Table 2. For decks A and B, participants received $1.00 with each card selection AND the penalty indicated for each respective deck in Table 2, and for decks C and D, participants received $0.50 with each card selection AND the penalty indicated for each respective deck in Table 2. Bechara et al. [26] used rewards and penalties with a base of 10, whereas we used a base of .10, however, the relative size of the rewards and penalties were the same as those used by Bechara et al. [26].

**Table 2:** The schedule of rewards and penalties in the four decks of the card task

Card Number	Deck A (+$1.00)‡	Deck B (+$1.00)‡	Deck C (+$.50)‡	Deck D (+$.50)‡
1				
2				
3	-$1.50		-$.25	
4				
5	-$3.00		-$.75	
6				
7	-$2.00		-$.25	
8				
9	-$2.50	-$12.50	-$.75	
10	-$3.50		-$.50	-$2.50
11				
12	-$3.50		-$.25	
13			-$.75	
14	-$2.50	-$12.50		
15	-$2.00			-$2.50
16				
17	-$3.00		-$.25	
18	-$1.50		-$.75	
19				
20			-$.50	
21		-$12.50		-$2.50
22	-$3.00			
23				
24	-$3.50		-$.50	
25			-$.25	
26	-$2.00		-$.50	
27	-$2.50			
28	-$1.50			
29			-$.75	
30			-$.50	
31	-$3.50			
32	-$2.50	-$12.50		-$2.50
33	-$2.50			
34			-$.25	
35			-$.25	
36				
37	-$1.50		-$.75	
38	-$3.00			
39			-$.50	
40			-$.25	
41		-$12.50	-$.50	-$2.50
42	-$3.00			
43				
44	-$3.50		-$.50	
45			-$.25	
46	-$2.00		-$.50	
47	-$2.50			
48	-$1.50			
49			-$.75	
50			-$.50	

‡ Note: Only the penalty amounts varied in each deck, which are indicated in the Table. The reward amounts were consistent for each deck, which were $1.00 for decks A and B and $.50 for decks C and D with each card selection

Participants were told that they would play a card game in which they had to select 100 cards, and that the purpose of the game was to maximize the amount of money they could win. They were told that on the back of each card in each deck, there was a reward or a reward and a penalty. They were to select cards one by one, and the examiner would give them the reward and collect the penalty after each card pull. Participants were loaned $20.00 at the beginning of the game. Participants were not given any cues or signals during the game about the content of each deck. Participant selections were recorded on a score sheet following the task. As play money was used, participants were told that they could win a $10 gift certificate to a popular local bookstore if they made a net gain in the game as an added incentive, as participants did not receive the actual amount of play money in the game.

The dependent measures were the total number of cards participants selected from each deck and the net amount of money participants had at the end of the game. At the end of the game, participants were asked about how the four decks differed to determine whether the participants realized the reward and penalty structure of the advantageous and disadvantageous decks. Specifically, they were asked: "Did you notice anything about the content of each of the decks?"

## Authors' Contributions

MT was involved in the theoretical conceptualization, data collection and analysis, and writing of the present manuscript. UJ was involved in the clinical assessment of the participants and feedback on the manuscript. RT was involved in the theoretical conceptualization, and provided ongoing input and feedback on the analysis and writing of this manuscript.
